# Ipsilateral femoral shaft and vertical patella fracture: a case report

**DOI:** 10.4076/1757-1626-2-7210

**Published:** 2009-07-30

**Authors:** Korhan Ozkan, Hakan Cift, Engin Eceviz, Adem Sahin, Ender Ugutmen

**Affiliations:** Department of Orthopaedics and Traumatology, Goztepe Research and Training HospitalFahrettin Kerim Gokay street. Kadikoy/ Istanbul post code 34722Turkey

## Abstract

**Introduction:**

A femoral shaft fracture with an ipsilateral patella fracture has been, to our knowledge, given only cursory attention in English-speaking literature.

**Case presentation:**

A 15 year old male patient had hitten by a car to his motorcycle came to emergency room and he had been operated for his femoral shaft freacture and vertical patellar fracture which was iniatally missed.

**Conclusion:**

To us it is vital to obtain CT scan of the patient’s knee if there is an ipsilateral femoral fracture with an ipsilateral knee effusion and a punction which reveals hematoma even in the absence of a fracture line seen in AP and lateral projections.

## Introduction

Femoral shaft fractures are commonly associated with injury to the ligaments of the knee joint [1-3]. However, a femoral shaft fracture with an ipsilateral patella fracture has been, to the best of our knowledge, given only cursory attention in English-speaking literature, Also, vertical patella fractures seems to be rare and it is difficult to see in the routine roenthgenographies of AP and lateral views [4]. An axial projection, such as sun rise or merchant views, may be valuable in suspected cases.

## Case presentation

**Figure 1.**
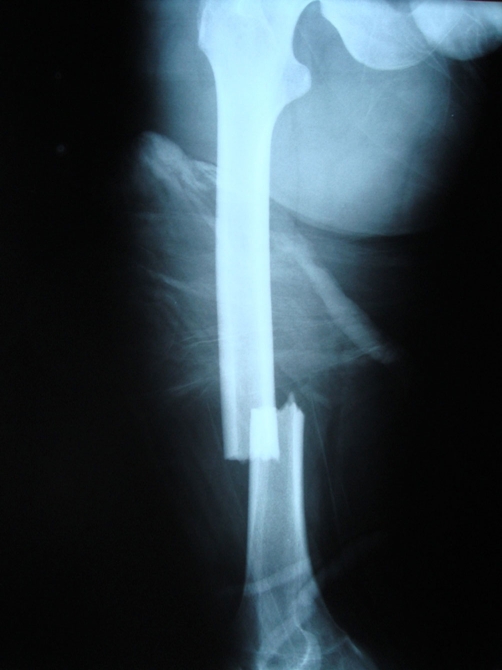
Preoperative AP roentgenography of femur shaft fracture.

**Figure 2.**
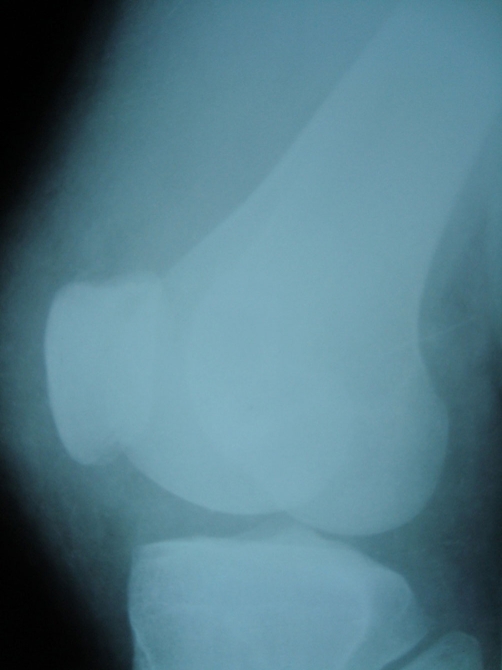
Preoperative lateral roentgenography of the knee.

**Figure 3.**
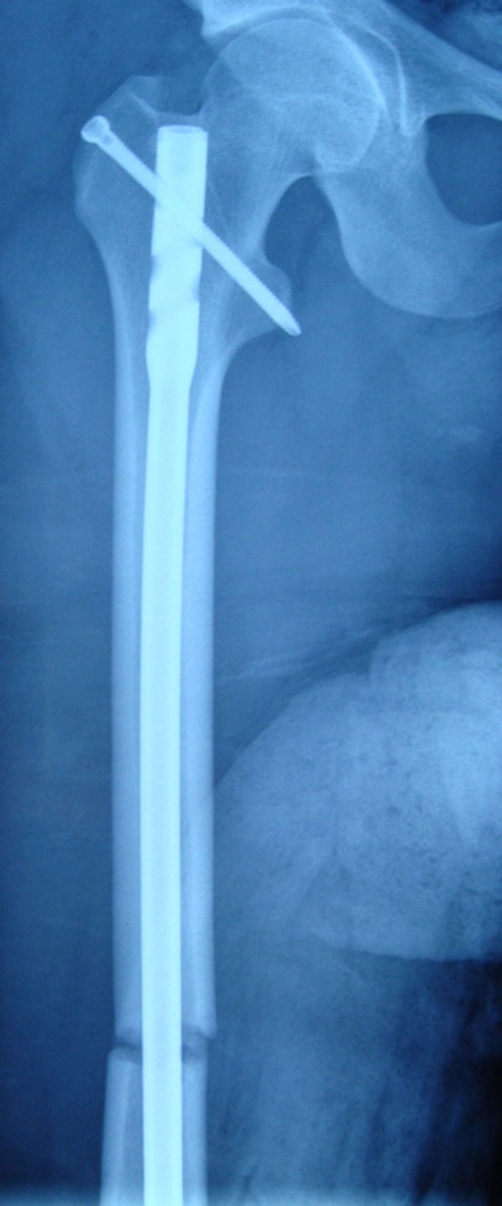
Postoperative AP roentgenography of femur shaft fracture.

**Figure 4.**
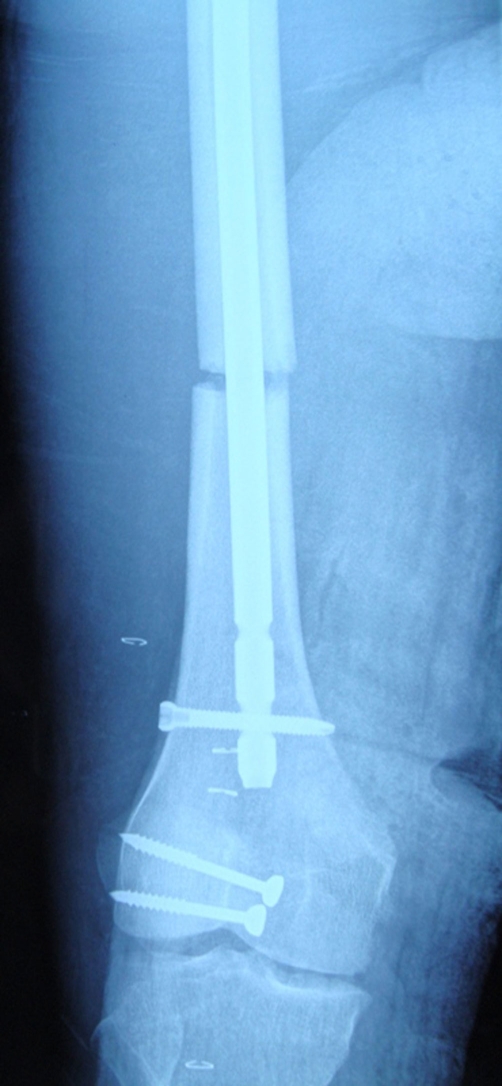
Postoperative AP roentgenography of patellar fracture and knee.

A 15 year old Caucasian male patient riding a motor cycle was hit by a car. He hit his knee to the bumper of the automobile and fell from the motorcycle. He was brought to the emergency by an ambulance. On examination, there was an injury in his left knee and ipsilateral thigh. Rest of the examination was normal. The x-rays of the femur showed us a displaced femoral shaft fracture (Figure 1). There was a mild effusion on his left knee but no fracture line was detected on the AP-Lateral radiographs (Figure 2). The patient was taken to emergency theatre and within two hours a locked intramedullar nailing (Trigen, Smith and Nephew, USA) was performed on the fracture table (Figure 3). After finishing the surgery we focussed our attention to left knee, because we noticed that swelling at the knee had increased. Re-examination of the knee was done which proved to be unyeilding except for the swelling. Aspiration of the knee was done which displayed hematoma with a fat droplets inside. Knee was again examined under image intensifier with AP, Lateral and this time in Merchant view as well. Merchant view revealed a vertical fracture of the patella. Through an anterior longitudinal incision, vertical fracture of the patella which was displaced more than 3 mm was reduced and fixed with 2 malleolar screws (Figure 4).

## Discussion

Ipsilateral femoral neck fractures occur in 2.5%-9% of all femoral shaft trauma cases. Of these, 78% result from motor vehicle accidents and 13% from other high-energy trauma such as falls [5].

Also, combined intertrochanteric and mid-shaft fractures of femur may occur in as many as 28% of all ipsilateral hip and femoral shaft fractures and are typically noncomminuted.

Patellar fractures are uncommon injuries and account for approximately 1% of all fractures [4]. Patellar fractures are classified according to both the mechanism of injury and the fracture morphology. The two major mechanisms of injury are direct and indirect trauma. Direct trauma frequently results in fracture comminution with little displacement. Indirect trauma results from rapid flexion of the knee against a fully contracted quadriceps resulting in displaced transverse fractures. However, most patella fractures occur as a result of a combination of direct and indirect trauma. Transverse fractures are the most common morphologic type of fracture pattern while vertical fractures occur rarely [4]. Being mostly undisplaced, the reported cases have almost always been treated non-operatively. But as a displaced fracture, stable fixation with two screws enabled us to start early range of motion exercises in our case.

It is now known that the patella plays a crucial role in the extensor mechanism of the knee. It functions to improve the lever arm of the extensor mechanism and increasing the magnitude of the quadriceps moment arm by nearly 30% at maximal extension. The patella is exposed to complex loading consisting of tensile, bending, and compressive forces of varying degrees depending on the position of the knee. Joint contact forces of 3.3 times the body weight may occur with stair climbing and up to 7.6 times body weight may occur with squatting activities. The contact area of the knee during range of motion is small, resulting in high contact stresses at the patellofemoral joint [6].

Vertical fractures of the patella are best seen on tangential or Merchant radiographies. A radiograph of the contralateral knee may be helpful in distinguishing acute fractures from bipartite patella which has been reported in 0.05 to 1.6% of the population [7]. Also, radiographies of the ipsilateral hip should be obtained in high energy direct trauma (i.e., dashboard injuries) to exclude proximal femoral or acetabular trauma. Magnetic resonance imaging may be used in young patients to diagnose a sleeve fracture. Computed tomographic (CT) examination may be valuable in suspected cases of vertical fracture.

Ipsilateral femoral and vertical patella fractures are extremely rare injuries and if the fracture of the patella is vertical it can be easily misdiagnosed. To us it is vital to obtain CT scan of the patient’s knee if there is an ipsilateral femoral fracture with an ipsilateral knee effusion and a punction which reveals hematoma even in the absence of a fracture line seen in AP and lateral projections.

## Conclusion

The most important diagnostic tool in vertical fractures of patella is physical examination of the patient. If there is an effusion at the knee and aspiration reveals hematoma with fat droplets inside, suspicion should rise about an osteochondral fracture even if nothing is detected on the radiographs in AP and lateral projections. In these cases it is crucial to obtain CT scan of the patient’s knee before surgery.

## Abbreviations

AP: Anteroposterior
CT: Computed tomography
